# Analysis of Clinical Outcomes of Pregnant Patients Treated With Nirmatrelvir and Ritonavir for Acute SARS-CoV-2 Infection

**DOI:** 10.1001/jamanetworkopen.2022.44141

**Published:** 2022-11-29

**Authors:** William M. Garneau, Kimberly Jones-Beatty, Michelle O. Ufua, Heba H. Mostafa, Sabra L. Klein, Irina Burd, Kelly A. Gebo

**Affiliations:** 1 Division of Hospital Medicine, Johns Hopkins University School of Medicine, Baltimore, Maryland; 2 Integrated Research Center for Fetal Medicine, Department of Gynecology and Obstetrics, Johns Hopkins University School of Medicine, Baltimore, Maryland; 3 Department of Pathology, Johns Hopkins University School of Medicine, Baltimore, Maryland; 4 Department of Molecular Microbiology and Immunology, Johns Hopkins University School of Medicine, Baltimore, Maryland; 5 Division of Infectious Diseases, Johns Hopkins University School of Medicine, Baltimore, Maryland

## Abstract

**Importance:**

Pregnant people are at increased risk of poor outcomes due to infection with SARS-CoV-2, and there are limited therapeutic options available.

**Objective:**

To evaluate the clinical outcomes associated with nirmatrelvir and ritonavir used to treat SARS-CoV-2 infection in pregnant patients.

**Design, Setting, and Participants:**

This case series included pregnant patients who were diagnosed with SARS-CoV-2 infection, received nirmatrelvir and ritonavir, and delivered their offspring within the Johns Hopkins Health System between December 22, 2021, and August 20, 2022.

**Exposures:**

Treatment with nirmatrelvir and ritonavir for SARS-CoV-2 infection during pregnancy.

**Main Outcomes and Measures:**

Clinical characteristics and outcomes were ascertained through manual record review.

**Results:**

Forty-seven pregnant patients (median [range] age, 34 [22-43] years) were included in the study, and the median (range) gestational age of their offspring was 28.4 (4.3-39.6) weeks. Medication was initiated at a median (range) of 1 (0-5) day after symptom onset, and only 2 patients [4.3%] did not complete the course of therapy because of adverse effects. Thirty patients (63.8%) treated with nirmatrelvir and ritonavir had a comorbidity in addition to pregnancy that could be a risk factor for developing severe COVID-19. Twenty-five patients [53.2%] delivered after treatment with nirmatrelvir and ritonavir. Twelve of these patients [48.0%] underwent cesarean delivery, 9 [75.0%] of which were scheduled. Two of 47 patients [4.3%] were hospitalized for conditions related to preexisting comorbidities.

**Conclusions and Relevance:**

In this case series, pregnant patients who were treated with nirmatrelvir and ritonavir tolerated treatment well, although there was an unexpectedly high rate of cesarean deliveries. The lack of an increase in serious adverse effects affecting pregnant patients or offspring suggests that clinicians can use this drug combination to treat pregnant patients with SARS-CoV-2 infection.

## Key Points

**Question:**

What outcomes are associated with nirmatrelvir and ritonavir for treatment of SARS-CoV-2 infection in pregnant patients?

**Findings:**

In this case series of 47 pregnant patients who were treated with nirmatrelvir and ritonavir, the medication was well tolerated without evidence of an increase in complications affecting birthing parents or their offspring. Approximately half of deliveries after treatment with nirmatrelvir and ritonavir were via cesarean delivery.

**Meaning:**

Results of this study suggest that pregnant patients with SARS-CoV-2 infection can be safely treated with nirmatrelvir and ritonavir.

## Teaser

This case series examines the outcomes experienced by pregnant patients prescribed nirmatrelvir and ritonavir to treat SARS-CoV-2 infection.

## Introduction

COVID-19 is associated with considerable morbidity and mortality, particularly among pregnant patients, which can affect the health of the developing fetus.^1^ Pregnant patients infected with SARS-CoV-2 have higher rates of severe COVID-19, enhanced immune response, higher preterm birth rates, and increased abnormal maternal vessels and thrombi in placental tissue compared with those without SARS-CoV-2 infection.^2,3,4,5^ Given the continued community transmission of SARS-CoV-2 despite vaccines being available, infection with SARS-CoV-2 will continue to affect pregnant patients, and treatment decisions pose challenges for maternal and fetal health clinicians.

The nirmatrelvir and ritonavir drug combination was granted an emergency use authorization by the US Food and Drug Administration (FDA) on December 22, 2021, for the treatment of mild to moderate COVID-19 in patients aged 12 years and older who are at high risk of progression to severe COVID-19.^6^ The drug is effective, acting as an inhibitor of the SARS-COV-2 main protease.^7^ The FDA states that there is inadequate knowledge of the effects of nirmatrelvir and ritonavir on the birthing parent and fetus to make a statement on its safety in pregnancy.^6^ Safety data for nirmatrelvir in rats and rabbits did not show either developmental toxic effects or detrimental effects on fertility.^8^ Data from the FDA show no increase in the risk of birth defects in pregnant individuals taking ritonavir.^9^

Nirmatrelvir and ritonavir showed no significant reduction in hospitalization and deaths in a subgroup analysis of patients with at least 1 risk factor for developing severe COVID-19.^10^ While there has been widespread adoption of nirmatrelvir and ritonavir as an oral option for outpatient treatment of SARS-CoV-2 infection, use of this drug combination in pregnancy is not well studied and may not be adequately used by patients and their clinicians due to lack of data in pregnant patients.

More than 130 000 new SARS-CoV-2 infections were reported daily in the United States as of August 2022.^11^ The safety of vaccination against SARS-CoV-2 for pregnant individuals has been reported previously; rates of vaccine uptake among pregnant people, however, is lower than expected, and vaccinated individuals remain at risk of SARS-COV-2 infection, particularly with variants of concern, despite vaccination.^12,13,14,15^ There are multiple effective therapies for outpatient treatment of acute SARS-CoV-2, including remdesivir, convalescent plasma, molnupiravir, and monoclonal antibodies. Yet each of these options has limitations. Remdesivir, monoclonal antibodies, and convalescent plasma must be administered intravenously. Molnupiravir is an oral treatment for COVID-19 treatment; its mechanism of action, however, introduces errors into RNA replication, and it is contraindicated in pregnancy due to harm to the developing fetus.^16^ The aim of the study was to evaluate outcomes in pregnant patients who were treated with nirmatrelvir and ritonavir within a large hospital system to assess the outcomes associated with this drug combination to treat SARS-CoV-2 infection in this patient population.

## Methods

This case series used the COVID-19 Precision Medicine Analytics Platform Registry (JH-CROWN), a database that includes outpatient and inpatient records for patients with SARS-CoV-2 within the Johns Hopkins Health System. The health system is located in Maryland and Washington, DC, and includes 6 hospitals and more than 40 outpatient facilities. The research was approved by the Core for Clinical Research Data Acquisition, which administers the JH-CROWN registry, and the Johns Hopkins Institutional Review Board, which waived the requirement for informed consent because only deidentified data were used. We followed the reporting guideline for case series in medicine.

Pregnant patients with a positive SARS-COV-2 test result within the Johns Hopkins Health System after FDA emergency use approval of nirmatrelvir and ritonavir from December 22, 2021, to August 20, 2022, were eligible for study inclusion. A query was written to identify patients with a diagnosis of pregnancy, a positive test result for SARS-CoV-2 between these dates, and a prescription for nirmatrelvir and ritonavir. Manual medical record review was performed on all medical records of patients who met these 3 criteria. Records were excluded if the patient was not pregnant, if there was no documentation of receipt of nirmatrelvir and ritonavir, or if records were duplicates. The medical records were reviewed at the time of the prescription, and subsequent notes were reviewed to assess patient outcomes and tolerance. Individual characteristics, including maternal age, gestational age, practitioner type, prepregnancy body mass index (BMI, calculated as weight in kilograms divided by height in meters squared), gravidity and parity, vaccination status, prepregnancy comorbidities, adverse effects after administration of nirmatrelvir and ritonavir, delivery date, and delivery complications, were collected using REDCap, version 12.0.16 (Vanderbilt University).^17,18^ Race and ethnicity were classified by self-report in the electronic medical record and were collected to characterize the differences in socioeconomic factors associated with access to care. Age was defined as the age when the patient was treated with nirmatrelvir and ritonavir. Vaccination status was defined as having had no vaccination, having received the initial vaccination series, or having received the initial vaccination series with 1 or 2 additional boosters. Comorbidities were collected from the Epic Systems electronic health record problem list on initiation of antenatal care. We reviewed the medical record for specific comorbidities based on the Centers for Disease Control and Prevention (CDC) and Infectious Diseases Society of America (IDSA) indications for outpatient treatment with nirmatrelvir and ritonavir including patient age 65 years or older, chronic kidney disease, diabetes, BMI of 25 or greater, chronic lung disease, sickle cell disease, cardiovascular disease or hypertension, use of immunosuppressing medications, neurodevelopmental disorders, and medical technological dependence.^19^

## Results

Forty-seven patients (median [range] age, 34 [22-43] years) were pregnant at the time they received nirmatrelvir and ritonavir from December 22, 2021, to August 20, 2022 (Figure), and the median (range) gestational age of their offspring was 28.4 (4.3-39.6) weeks. The median (range) time from symptom onset to receipt of medication was 1 (0-5) day. The baseline characteristics of pregnant patients receiving nirmatrelvir and ritonavir are presented in Table 1.

**Figure.**
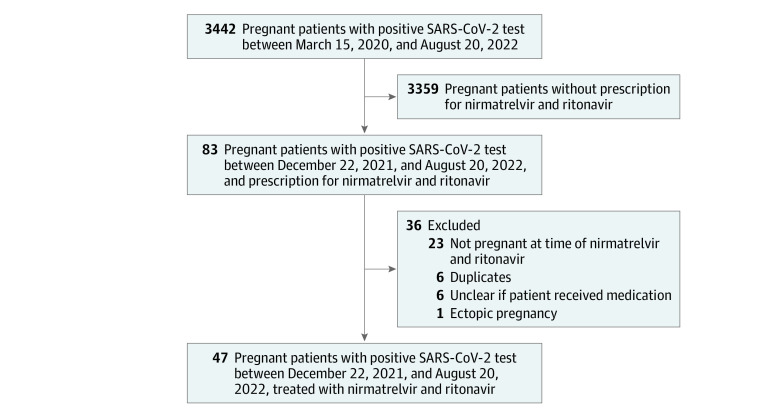
Cohort Flowchart

**Table 1.** Baseline Characteristics of Pregnant Patients Receiving Nirmatrelvir and Ritonavir

Characteristic	Patients, No. (%) (n = 47)
Age, median (range), y	34 (22-43)
Race	
Asian	4 (8.5)
Black or African American	8 (17.0)
White	31 (66.0)
Other^a^	2 (4.3)
Unknown	2 (4.3)
Ethnicity	
Hispanic or Latino	5 (10.6)
Not Hispanic or Latino	41 (87.2)
Unknown	1 (2.1)
Gestational age at treatment, median (range), wk	28.4 (4.3-39.6)
Trimester of pregnancy at treatment	
First	4 (8.5)
Second	16 (34.0)
Third	27 (57.4)
Prepregnancy BMI (n = 46)^b^	
<18.5	0
18.5-24.9	22 (47.8)
25.0-29.9	14 (30.4)
≥30.0	10 (21.7)
Gravidity	
1	8 (17.0)
2	14 (29.8)
3	8 (17.0)
>3	17 (36.2)
Parity	
0	16 (34.0)
1	21 (44.7)
2	5 (10.6)
>2	5 (10.6)
Prepregnancy medical comorbidities	
Mental health disorder	21 (44.7)
Abnormal Papanicolaou test	13 (27.7)
Obesity (BMI ≥30)	12 (25.5)
Anemia	12 (25.5)
Asthma	11 (23.4)
Gastrointestinal disorder^c^	8 (17.0)
Other^d^	6 (12.8)
Diabetes	5 (10.6)
Substance use	5 (10.6)
Thyroid disorder	5 (10.6)
Venous thromboembolism	3 (6.4)
Hypertension	3 (6.4)
Hyperlipidemia	2 (4.3)
Cardiac arrhythmia	2 (4.3)
Inflammatory bowel disease	2 (4.3)
Sickle cell disease	1 (2.1)
Preeclampsia	1 (2.1)
Antiphospholipid syndrome	1 (2.1)
Indications for receipt of nirmatrelvir and ritonavir^e^	
BMI ≥25	24 (51.1)
Pregnancy alone	17 (36.2)
Chronic lung disease	10 (21.3)
Diabetes	5 (10.6)
Cardiovascular disease or hypertension	4 (8.5)
Sickle cell disease	1 (2.1)
COVID-19 vaccination status	
No vaccination	7 (14.9)
Received initial vaccination series	16 (34.0)
Received initial vaccination series and 1 booster	21 (44.7)
Received initial vaccination series and 2 boosters	3 (6.4)

Abbreviation: BMI, body mass index (calculated as weight in kilograms divided by height in meters squared).

^a^ No additional information was available in the data about the other races.

^b^ Data were missing for 1 patient.

^c^ Gastrointestinal disorders include gastroesophageal reflux disease, gastritis, nausea, and irritable bowel syndrome.

^d^ Other prepregnancy medical comorbidities include carotid artery dissection, endocarditis, vertebral dissection, thymoma, postural orthostatic tachycardia syndrome, and idiopathic thrombocytopenic purpura.

^e^ Based on guidelines from the US Centers for Disease Control and Prevention and the Infectious Diseases Society of America.

Twenty-seven patients (57.4%) received nirmatrelvir and ritonavir in the third trimester of pregnancy, 16 (34.0%) in the second trimester, and 4 (8.5%) in the first trimester. Forty patients (85.1%) had received some vaccination: 16 (34.0%) had received the initial vaccination series, 21 (44.7%) had received the initial vaccination series and 1 booster, and 3 (6.4%) had received the initial vaccination series and 2 boosters. Forty-three patients (91.5%) had any comorbidity, with mental health disorder (21 [44.7%]) being the most commonly reported. Thirty patients (63.8%) had a comorbidity other than pregnancy that qualified as a CDC and IDSA indication for outpatient treatment with nirmatrelvir and ritonavir, with the most common indication being a BMI of 25 or greater (24 [51.1%]).

All patients were either treated for COVID-19 as outpatients or were treated while hospitalized for reasons other than COVID-19. Thirty-seven prescriptions (78.7%) were written by obstetricians and gynecologists, and 10 (21.3%) were written by midlevel practitioners (Table 2). Twelve prescriptions (25.5%) were filled in May 2022, 17 (36.2%) in June 2022, 12 (25.5%) in July 2022, and 6 (12.8%) in August 2022. Viral sequencing data were available for 5 patients (10.6%) whose testing was performed within the Johns Hopkins Health System. Four of the samples (80.0%) were identified as Omicron (BA.4, BA.2, BA.5, unassigned) and 1 (20%) as Delta (21I).

**Table 2.** Patient Outcomes

Characteristic	Patients, No. (%) (n = 47)
Type of practitioner ordering nirmatrelvir and ritonavir	
Obstetrician and gynecologist	37 (78.7)
Midlevel practitioner	10 (21.3)
Time from symptom to receipt of medication, median (range), d	1 (0-5)
Month of nirmatrelvir and ritonavir prescription	
December-April	0
May	12 (25.5)
June	17 (36.2)
July	12 (25.5)
August	6 (12.8)
Patient discontinued medication due to adverse effects	2 (4.3)
Patient hospitalized after taking nirmatrelvir and ritonavir	2 (4.3)
Patient delivered after taking nirmatrelvir and ritonavir	25 (53.2)

Two patients (4.3%) were hospitalized during pregnancy after treatment with nirmatrelvir and ritonavir: 1 for vomiting and dehydration in the setting of preexisting hyperemesis gravidarum and 1 for persistent cough in the context of sickle cell crisis; this patient was identified as having placenta accreta and experienced postpartum hemorrhage. Two patients (4.3%) discontinued the medication before completing treatment due to adverse effects. Three patients (6.4%) developed gestational hypertension. One patient (2.1%) developed excessive fetal growth and polyhydramnios. One patient (2.1%) developed oligohydramnios. One patient (2.1%) with a history of bicornuate uterus and multiple children with genetic disorders experienced the loss of a twin pregnancy at 12 weeks. A fetus that was the product of the pregnant patient’s in-vitro fertilization at age 40 years was noted to have absent ductus venosus as well as thickened nuchal fold. One fetus developed intrauterine growth restriction and syndactyly and was noted to have cystic hygroma before nirmatrelvir and ritonavir exposure. Twenty-five patients (53.2%) delivered after treatment with nirmatrelvir and ritonavir. Patient outcomes are summarized in Table 2.

Twelve of the 25 patients (48.0%) who delivered after treatment underwent cesarean delivery; 9 of these (75.0%) were scheduled. One unplanned cesarean delivery was performed during the birth of twins in which arrest of labor occurred after the first vaginal birth, 1 was in the context of fetal intolerance of labor, and 1 was in the context of oligohydramnios and breech presentation. Delivery outcomes are summarized in Table 3.

**Table 3.** Characteristics of Deliveries After Patient Receipt of Nirmatrelvir and Ritonavir

Maternal age, y	Gravidity	Parity	Trimester patient received nirmatrelvir and ritonavir	Vaccination status	Comorbidities^a^	Risk factors other than pregnancy for developing severe COVID-19^b^	BMI	Cesarean delivery	Indication for cesarean delivery	Newborn birth weight, g
34	2	0	3	Vaccinated	Preeclampsia, abnormal Papanicolaou test	BMI ≥25	25.7	No	NA	3620
30	4	2	3	Not vaccinated	Asthma, mental health disorder, obesity, substance use	BMI ≥25, chronic lung disease	31.7	Yes	Scheduled repeat cesarean delivery	3150
27	>5	2	3	Vaccinated	Mental health disorder, obesity	BMI ≥25	30.7	No	NA	3435
33	4	1	3	Vaccinated with 1 booster	Diabetes, thyroid disorder	BMI ≥25, diabetes	28.1	Yes	History of breech presentation	3270
43	>5	2	3	Vaccinated	Mental health disorder, obesity, history of venous thromboembolism, anemia	BMI ≥25	44.3	Yes	History of breech presentation	3657
22	2	1	3	Vaccinated	Anemia	None	24.8	No	NA	3910
39	>5	0	3	Vaccinated with 1 booster	Mental health disorder, sickle cell disease, anemia	Sickle cell disease	21.6	Yes	Planned due to placenta accreta	2410
33	1	0	2	Vaccinated with 1 booster	Obesity	BMI ≥25	40.4	No	NA	3080
39	2	1	2	Vaccinated with 1 booster	History of venous thromboembolism, abnormal Papanicolaou test, antiphospholipid syndrome	None	24.1	No	NA	3070
41	1	1	3	Vaccinated with 1 booster	Diabetes, abnormal Papanicolaou test	BMI ≥25, diabetes	27.9	Yes	Elective primary cesarean delivery	3033
38	5	4	3	Vaccinated with 1 booster	Anemia	None	23.7	No	NA	2940
36	5	3	3	Vaccinated	Diabetes, obesity	BMI ≥25, diabetes	37.5	Yes	Scheduled cesarean delivery	3770
30	>5	1	3	Not vaccinated	Mental health disorder, cardiac arrhythmia, abnormal Papanicolaou test, anemia	Cardiovascular disease	19.6	No	NA	3670
30	1	0	3	Vaccinated	Hyperlipidemia, obesity, anemia	BMI ≥25	30.4	No	NA	2540
34	4	1	3	Vaccinated with 1 booster	Asthma, abnormal Papanicolaou test, anemia	BMI ≥25, chronic lung disease	26.6	Yes	Scheduled repeat cesarean delivery	2840
35	3	0	3	Vaccinated with 1 booster	Anemia	None	22.9	Yes	Fetal intolerance of labor	3440
30	2	1	3	Vaccinated	Abnormal Papanicolaou test, anemia, carotid artery dissection	None	23.1	No	NA	3290
27	2	1	3	Not vaccinated	Asthma	BMI ≥25, chronic lung disease	25.4	Yes	Cesarean delivery due to arrest of labor	3000
33	2	1	3	Vaccinated with 1 booster	Mental health disorder	None	19.5	No	NA	3300
26	5	4	3	Vaccinated	Asthma, mental health disorder, abnormal Papanicolaou test, anemia	BMI ≥25, chronic lung disease	26.7	Yes	Scheduled repeat cesarean delivery	2190
38	2	1	3	Vaccinated with 2 boosters	Diabetes, inflammatory bowel disease	Diabetes	24.9	Yes	Scheduled repeat cesarean delivery	4650
34	>5	1	3	Not vaccinated	Mental health disorder, substance use disorder, endocarditis	Cardiovascular disease	24.0	Yes	Oligohydramnios, breech presentation	3350
30	1	0	3	Not vaccinated	Asthma, mental health disorder, inflammatory bowel disease	Chronic lung disease	22.6	No	NA	3040
27	>5	2	3	Vaccinated	Mental health disorder, obesity	BMI ≥25	26.9	No	NA	3220
39	2	1	3	Vaccinated	Mental health disorder, abnormal Papanicolaou test	None	24.3	No	NA	3360

Abbreviations: BMI, body mass index (calculated as weight in kilograms divided by height in meters squared); NA, not applicable.

^a^ Obesity is defined as having a BMI of 30 or greater.

^b^ Based on guidelines from the US Centers for Disease Control and Prevention and the Infectious Diseases Society of America.

## Discussion

This study has several important findings. First, the nirmatrelvir and ritonavir combination was well tolerated and did not pose an immediate threat to the birthing parent or fetus in this study. In addition, 63.8% of pregnant patients who received nirmatrelvir and ritonavir were prescribed the drug combination for an indication other than pregnancy, and 78.7% of prescriptions were written by obstetricians and gynecologists. Most patients who received nirmatrelvir and ritonavir were vaccinated, and relatively few Black patients were included in the cohort, suggesting that disparities in vaccine uptake may also be reflected in the use of nirmatrelvir and ritonavir as a therapy in pregnancy.

Only 2 patients discontinued nirmatrelvir and ritonavir due to adverse effects, and no complications were associated with the drug. This finding was replicated in another descriptive study from the University of Connecticut of 7 pregnant patients treated with nirmatrelvir and ritonavir who all experienced symptomatic improvement and no adverse pregnancy outcomes.^20^ In accordance with our findings, 85.7% of patients in that study were vaccinated before nirmatrelvir and ritonavir use; racial demographic characteristics were not reported. In contrast to our findings, the 3 deliveries in that study were vaginal births. The present study had a relatively high rate of cesarean deliveries in patients who received nirmatrelvir and ritonavir and delivered.

The CDC and IDSA outpatient treatment guidelines include pregnant patients among populations at higher risk for developing severe COVID-19 and who are eligible to use nirmatrelvir and ritonavir.^19,21^ This recommendation is supported by the Society for Maternal-Fetal Medicine.^22^ In the present study, prescriptions for pregnant patients did not begin until May 2022 and increased in June 2022. Seventy-nine percent of prescriptions were from obstetricians, which likely reflects the diverse roles of obstetricians and gynecologists in treating their pregnant patients. In the present study, we found no serious adverse effects associated with nirmatrelvir and ritonavir in pregnant patients. Because COVID-19 will continue to present in a variety of settings, it is important for other health care practitioners to use this drug combination in this high-risk population.

Although Johns Hopkins Health System cares for a diverse patient population, the patients in this study were of advanced age, and few were of racial or ethnic minority groups. It was unclear if all pregnant patients with SARS-CoV-2 were offered treatment or if rates of refusal differed by age group. Future studies will need to be conducted to evaluate disparities in receipt of treatment.

### Limitations

There are several limitations to this study, including the use of data from a single health system and a small population studied with mild illness. However, to our knowledge, this is the largest study to date in this population and found no serious adverse effects to the patients or their fetuses. The rise of at-home COVID-19 testing and use of registries designed for hospitalized patients means some patients were not included due to unrecorded testing and lack of standardized outpatient evaluation tools. Another limitation was the short follow-up period. Only 25 of 47 patients had undergone delivery at the time of this report. Treatment effects may not be apparent at delivery, and long-term effects of treatment will need to be followed up in both the birthing parents and children.

## Conclusions

Findings of this case series support the safety and effectiveness of nirmatrelvir and ritonavir in pregnant patients with acute SARS-CoV-2. Although there are multiple effective therapies for outpatient treatment of acute SARS-CoV-2, all are either intravenous (remdesivir, convalescent plasma, and monoclonal antibodies) or contraindicated in pregnancy (molnupiravir). Nirmatrelvir and ritonavir can be easily used as a first-line outpatient treatment for pregnant patients. Future larger studies will be needed to evaluate for rare complications in neonates or birthing parents who are treated with this medication. The serious risk of morbidity from SARS-CoV-2 among pregnant patients is further justification for practitioners to encourage vaccination and use of nirmatrelvir and ritonavir to minimize the risk to pregnant patients and their fetuses.
